# Thinking of Me or Thinking of You? Behavioral Correlates of Self vs. Other Centered Worry and Reappraisal in Late-Life

**DOI:** 10.3389/fpsyt.2022.780745

**Published:** 2022-06-22

**Authors:** Akiko Mizuno, Helmet Talib Karim, Jordyn Newmark, Faiha Khan, Matthew Joseph Rosenblatt, Alyssa M. Neppach, MaKayla Lowe, Howard Jay Aizenstein, Douglas S. Mennin, Carmen Andreescu

**Affiliations:** ^1^Department of Psychiatry, University of Pittsburgh, Pittsburgh, PA, United States; ^2^Department of Bioengineering, University of Pittsburgh, Pittsburgh, PA, United States; ^3^Department of Neuroscience, University of Pittsburgh, Pittsburgh, PA, United States; ^4^Department of Psychology, University of Pittsburgh, Pittsburgh, PA, United States; ^5^Department of Counseling and Clinical Psychology, Teachers College, Columbia University, New York, NY, United States

**Keywords:** worry, anxiety, generalized anxiety disorder, late-life, self-referential processing

## Abstract

Psychotherapeutic approaches in late-life anxiety have limited effect on reducing worry severity. The self-referential processing of worry contents (self- vs. other-focused worry) and reappraisal styles (internal vs. external locus of control) are important elements in psychotherapy, but little is known about these processes in late-life. We aimed to characterize severe worry from a self-referential processing perspective. We recruited 104 older adults with various levels of worry and used a personalized task to induce and reappraise worry. We analyzed the association between (1) worry severity/frequency for worry content (self- or other-focused) and (2) for reappraisal style (internal vs. external locus of control) with clinical inventories measuring anxiety, worry, depression, rumination, neuroticism, emotion regulation strategies, perceived stress, and physical illness burden. Higher self-worry severity was associated with higher scores of clinical inventories of worry, depression, perceived stress, and neuroticism, whereas other-worry severity did not show any association. Greater self-worry frequency was associated with higher medical burden. External locus of control in reappraisal statements was associated with lower worry severity in men. Overall, more severe and frequent self-focused worry was associated with a greater psychological and physiological burden. These results are useful in tailoring psychotherapy for older adults with severe worry.

## Introduction

Anxiety disorders are among the most common mental health disorders in older adults, with prevalence rates ranging between 1.2 to 15% (1), and yet they are often undiagnosed and untreated (2). Generalized Anxiety Disorder (GAD) is characterized by excessive, persistent, and difficult-to-control worry. Severe and uncontrollable worry is not only associated with impairments in daily function and reduced quality of life, but also with accelerated cognitive decline (3, 4), increased risk of brain aging (5), and increased risk of stroke and other cardiovascular events (6–8). Despite such a paramount public health impact on the global aging population, the mechanisms underlying worry in older adults are poorly understood and understudied.

Effective treatment is crucial considering the health implications associated with untreated anxiety disorder in the elderly (9). Treatment of GAD in late-life is less effective compared to younger adults and has limited therapeutic effects on worry severity (10). Cognitive reappraisal–a well-known adaptive emotion regulation strategy—is a core component of cognitive-behavioral therapy. The limited effectiveness of this strategy in older adults may be accounted for by multiple factors including age-related structural changes in key brain regions [e.g., dorsolateral pre-frontal cortex (11)] but also by neuropsychological reasons such as switching cost [i.e., the required high cognitive load to execute cognitive reappraisal (12, 13)], or the lack of familiarity with cognitive reappraisal strategies. Another factor that may contribute could be the qualitatively different content of worry in older adults compared to younger adults. Significant life changes (retirement, caregiving, declining physical and cognitive states) induce late-life specific worry themes. Older adults with GAD worry more about personal health but less about work/school compared to both younger adults with GAD (14) and older adults without GAD (i.e., subsyndromal and healthy controls) (15). Although both studies indicate that worrying about one's own health is a characteristic of late-life GAD, their conclusions were inconsistent: one concluded that worrying about personal health is a pathological feature (15) whereas another concluded that it was a common non-pathological aspect of worry among older adults (14). On the other hand, both studies agreed that worrying about family matters is normative in late-life (e.g., health of significant others).

This notion of excessive self-focused worry (i.e., *one's own* health), also called negative self-referential processing (16), has been repeatedly reported in anxiety disorders (17–19). The present analysis approached worry as a transdiagnostic construct across mood disorders and aimed to characterize the pathological form of worry from a self-referential processing perspective in late-life. In particular, we focused on two types of worry: worry regarding oneself (self-worry) and worry regarding others (other-worry). Thus, self-worry refers to worrisome themes in which the self is the object of worry (e.g., “I am worried that my cancer will come back”). In other-worry, the object of worrisome thoughts is others (e.g., “I am worried that my daughter will not find the right person”).

An important variable in self- vs. other-related processing in worry and anxiety, as well as cognitive reappraisal, is the locus of control. Locus of control refers to an individual's belief of internal or external control over experiencing outcomes (20, 21). In anxiety disorders, a perceived ability to control aversive events (i.e., emotions and bodily reactions) is known as anxiety-control belief. Rapee (22) and Barlow (23) posited that a lack of perceived control is considered as a central experience of this disorder. An internal locus of control has been previously associated with favorable outcomes, such as less perceived stress and better functional coping skills (24). Among individuals with anxiety disorders, external locus of control has been associated with psychopathological characteristics, including higher levels of state anxiety, more depressive symptoms, and fatigue (25). The external locus of control is a recognized risk factor in late-life anxiety (26). The locus of control is usually measured by a questionnaire (e.g., Internal-External Control Scale) (20), which can estimate participants' general believes of how strongly they have control over the outcomes of events. In this study, however, we analyzed the locus of control (internal vs. external) in participants' personalized reappraisal statements to estimate item-based locus of control corresponding to their own specific worry themes. For example, to alleviate worry about getting cancer, some cognitive reappraisals like, “I already do cancer screenings with my doctor to keep checks on my health,” utilize an internal locus of control (i.e., internal reappraisal), but some like, “My husband will take care of me if I get too sick to take care of myself,” utilize an external locus of control (i.e., external reappraisal).

To understand the potentially differential effect of self vs. other in worry content and locus of control, we conducted an analysis in which we interviewed participants to get 16 personalized worry (induction) statements and eight corresponding reappraisal statements. Participants rated the severity of worry for each personalized worry statement as well as their worry severity after each reappraisal statement. This task was developed and validated for a functional Magnetic Resonance Imaging (fMRI) in-scanner worry induction and reappraisal protocol (27–29). In this analysis, we focused on the behavioral data related to:

1) whether the object of reported worry statements was themselves (self-focused worry) or somebody else (other-focused worry),
2) whether reappraisal statements reflected internal or external locus of control, and
3) the reported severity of worry following each of the worry and reappraisal statements.

We examined how these features were associated with clinical measures of worry severity, anxiety, depression, perceived stress, rumination, neuroticism, emotion regulation, and physical illness burden. Based on the previous reports of negative self-referential processing in anxiety disorder (e.g., excessive self-focus) (16–19), we hypothesized that greater severity and frequency of self-focused worry would be associated with psychopathologic characteristics of anxiety disorders measured by self-report assessments (e.g., worry, depression, self-perceived stress). Given the anxiogenic effect of external locus-of-control (25), we also hypothesized that external reappraisal would be associated with more severe self-reported worry and psychopathological features of anxiety. It must be noted that assessing the relationship between worry severity of personalized worry statements (behavioral data from the task, item 3 above) and a clinical measure of worry [measure by Penn State Worry Questionnaire (PSWQ)] may appear tautological or autocorrelated; however, examining this association allows us to test our hypothesis (mentioned above) because these two variables reflect the different constructs: our measure of worry severity is an acute index for the severity of participants' specific worries which can be separately computed for self- and other-focused worry (i.e., a degree of self-referential processing), whereas PSWQ score reflects a chronic degree of overall severity of worry.

## Materials and Methods

### Participants and Study Design

Participants were recruited as part of the Functional Neuroanatomy Correlates of Worry in Older Adults (FINA) study (R01 MH108509). To investigate worry as a transdiagnostic construct across disorders, we recruited participants (*n* = 104) who were 50 years and older who had worry along a wide spectrum, such that the degree of worry severity was relatively normally distributed. To capture the earliest periods of severe worry in late life, we included participants over 50 years old. We recruited these participants regardless of clinical diagnosis including anxiety disorders (GAD, panic disorder, social phobia, etc.) and mood disorders (e.g., major depressive disorder, persistent depressive disorder, or unspecified depressive disorder), including 30.8% with GAD, 3.8% who were non-GAD, and/or 8.7% with MDD. Exclusion criteria were 1) diagnosis with autism spectrum disorders, intellectual development disorder, any form of psychosis or bipolar disorder, a personality disorder, major neurocognitive disorder (e.g., dementia), cerebrovascular accident, multiple sclerosis, vasculitis, or significant head trauma, 2) a Modified Mini-Mental State (30) score <84, 3) increased suicide risk as assessed by the primary psychiatrist on the study (CA), 4) use of antidepressants within the last 5-14 days (participants were allowed to washout), a history of drug/alcohol abuse within last 6 months, or use of high doses of benzodiazepines (greater than equivalent to 2 mg of lorazepam), 5) uncorrected vision problems that would preclude neuropsychological testing, 6) below sixth grade level of reading, or 7) ferromagnetic objects in body, claustrophobia, or too large to fit in MRI scanner. While we did not analyze any MRI data here, the data used in this study was obtained as part of a neuroimaging study.

When appropriate, participants underwent an adequate washout on antidepressants determined by the primary psychiatrist on the study (CA). For fluoxetine, the washout interval was 6 weeks. Participants who were prescribed low dose psychotropics for pain, sleep disturbances, and/or medical conditions were allowed in most circumstances. The following common antidepressants were allowed at particular doses due to medical reasons: amitriptyline (50 mg/day), doxepine (50 mg/day), trazodone (100 mg/day), and imipramine (50 mg/day). Participants were recruited from the Pittsburgh area via Pitt+Me (website resource from the university), in-person recommendations, flyers around the city, and radio/television announcements. This study was approved by the University of Pittsburgh Institutional Review Board. All participants gave written informed consent prior to participating in the study.

### Assessments

Along with demographic information (age, sex, race, and education), we assessed the following: worry (PSWQ, Penn State Worry Questionnaire) (31), anxiety (HARS, Hamilton Anxiety Rating Scale) (32), depression (MADRS, Montgomery-Åsberg Depression Rating Scale) (33), rumination subscale (RSQ, Response Style Questionnaire) (34), neuroticism subscale (NEO-FFI, NEO-Five-Factor Inventory) (35), stress (PSS, Cohen's Perceived Stress Scale) (36), and the habitual use of cognitive reappraisal and suppression subscale (ERQ, Emotion Regulation Questionnaire) (37). We also collected data on illness severity (CIRS-G, Cumulative Illness Rating Scale for Geriatrics) (38).

### Worry Task

We conducted a personalized worry task in the MRI scanner—fMRI results are not presented here, but we have published on a similar task in the past (27–29). Participants were first interviewed by trained research assistants to elicit specific worry themes. Participants were aided to come up with 16 personalized worry statements that induced varying levels of worry severity. For eight such statements, they were aided in coming up with statements that helped reappraise that worry using cognitive reappraisal strategies. For instance, one such statement was “Worry that my grandson will get hit by a car while delivering.” The reappraising statement for this was “Pittsburgh is becoming more and more bike friendly.” These personalized worry statements were shown in a random order to participants for 25 sec, during the last 15 sec they were asked to rate their worry (“Rate your worry now”) from one (not worried at all) to five (extremely worried). The eight reappraising statements were always preceded by their paired worry statements. There were also eight neutral statements, but these were not analyzed here. Each block was followed by a 10 sec interval of fixation. The task was coded in PsychToolbox-3 (39).

### Measures of Self vs. Other Worry and Reappraisal

We randomly sorted each worry statement (across session and participant) and rated them as either the object of worry being self [“self(-focused) worry”] or someone else [“other(-focused) worry”]. An example of a “self-worry” is “Worry that you can't pay back the home equity loan.” In this example, the participant is worried about their home equity loan payments. An example of an “other-worry” statement is “Worry about your grandson becoming disabled.” In this example, the participant is worried about their grandson and not themselves.

We then randomly sorted each reappraisal statement (across session and participant) and rated them as either having an internal locus of control or external locus of control—the corresponding worry statement was shown along with it. For example, one participant had the following worry induction: “Worry that your sister will not be as active as she used to be” —and their reappraisal of this had an internal locus of control “I get my sister out of the house and take her to her grandson's baseball games to help her stay active.” In this example, the participant has taken it upon themselves to ensure that the object of their worry (their sister) stays active. Another participant had the following worry induction: “Worry that your neighbor's surgery will get messed up.” Their reappraisal of this had an external locus of control “Your neighbor's parents are retired and could help her.” In this example, the participant relies on someone else (their neighbor's parents) to help reappraise their worry.

All worry statements and reappraisal statements were rated as self/other by three raters: JN, AN, and ML. JN was given significant training and rated all worry and reappraisal statements as either self/other/not applicable (NA). Two student raters (AN and ML) also rated the statements following training. We computed the inter-rater reliability by using Fleiss Kappa and root package for bootstrapping (10,000) in R (“irr” and “root” packages), and the Fleiss Kappa (k) = 0.80, 95% CI [0.78, 0.82] (z = 74.8, *p* < 0.001) indicated a strong agreement (40, 41). Statements that were disagreed on were adjudicated by a fourth reviewer (AM).

We then computed six outcome measures for each participant in MATLAB 2018b (Natick, MA: The MathWorks Inc.):

1. **self-worry severity**—average worry rating among 16 worry statements rated as self-focused;
2. **other-worry severity**—average worry rating among 16 worry statements rated as other-focused;
3. **self-worry frequency**—frequency of self-focused worry among 16 worry statements;
4. **worry severity with internal reappraisal**—average of eight worry ratings after reappraisal statements rated as internal locus of control (i.e., an index of effectiveness of internal locus of control in reappraisal);
5. **worry severity with external reappraisal**—average of eight worry ratings after reappraisal statements rated as external locus of control (i.e., an index of effectiveness of external locus of control in reappraisal);
6. **internal reappraisal frequency**—frequency of internal locus of control among eight reappraisal statements;

Thus, for worry severity with internal and external reappraisal, greater average values indicate less effective reappraising statements as these ratings follow the reappraising statements. Therefore, a high value on worry severity with internal reappraisal indicates that for that participant, the reappraisal that utilized an internal locus of control was not effective.

We conducted statistical analyses to understand the factors associated with each of these measures (details below). For two frequency variables (self-worry frequency, internal reappraisal frequency), we coded the “self” statement as +1 and the “other” statement as −1 and computed the average (which was done for worry as well as reappraisal separately).

### Statistical Analysis

We conducted all statistical analyses in R [R Core (42)]. We first reviewed the patterns of missing data visually with a plot of missing value patterns and assumed that they were missing at random (i.e., no monotonicity) (Table 1). We then conducted multiple imputations using the random forest approach of the ‘mice’ package in R (43). Briefly, this approach uses random forest regression/classification to determine missing values by predicting each variable using a random forest model that resamples (bootstrapping with replacement) and chooses a subset of variables and generates many decision trees. All variables were included in the permutation along with the outcome measures (44).

**Table 1:** Characteristics of the sample.

**Variable Name**	**Total sample (*****n*** **=** **104)**			**Number Missing**	**Imputed Mean (Std.)**
	**Mean**	**Std**.	**Min, Max**		
Age, years	61.34	8.34	50, 80	0	N/A
Sex, number female	68 (65.4%) F			0	N/A
Race, W/B/HPI/MR	86 (82.7%)/ 16 (15.4%)/1(1.0%)/ 1 (1.0%)			0	N/A
Education, years	15.60	2.51	9, 22	2	16 (3)
Worry (PSWQ)	49.92	14.85	21, 80	1	50.06 (14.84)
Anxiety (HARS)	8.74	7.16	0, 38	3	8.78 (7.21)
Depression (MADRS)	8.39	8.27	0, 38	6	8.70 (8.58)
Rumination (RSQ)	38.16	13.00	22, 84	6	38.68 (13.50)
Neuroticism (NEO–FFI Subscale)	20.23	10.30	2, 44	11	20.69 (10.39)
Perceived Stress (PSS)	15.72	8.30	0, 34	8	16.06 (8.47)
Reappraisal (ERQ Subscale)	19.82	7.49	6, 42	8	29.92 (7.41)
Suppression (ERQ Subscale)	13.79	5.37	2, 25	8	13.77 (5.45)
Cumulative Illness (CIRS–G)	4.05	3.40	0, 15	4	4.12 (3.38)
Self–Worry Severity	2.97	1.10	1.00, 5.00	0	N/A
Other–Worry Severity	2.93	1.18	1.00, 5.00	0	N/A
Self–Worry Frequency	0.06	0.69	−1.00, 1.00	0	N/A
Self–Worry Counts (/16 statements)	9.11	4.74	1, 16	0	N/A
Other–Worry Counts (/16 statements)	8.89	4.85	1, 16	0	N/A
N/A Counts (/ 16 statements)	1.81	0.88	1, 4	0	N/A
Worry Severity w/ Internal Reappraisal	2.28	0.85	1.00, 4.50	0	N/A
Worry Severity w/ External Reappraisal	2.12	0.91	1.00, 4.17	0	N/A
Internal Reappraisal Frequency	0.05	0.59	−1.00, 1.00	0	N/A
Internal Reappraisal Counts (/8 statements)	4.49	2.11	1, 8	0	N/A
External Reappraisal Counts (/8 statements)	4.40	1.95	1, 8	0	N/A
N/A Counts (/ 8 statements)	1.25	0.50	1, 2	0	N/A

*Means and standard deviations are reported unless otherwise noted. Means and standard deviations for both the original data and imputed values (see number of missing data) are reported. N/A – not applicable or not imputed. Race, W–White, B – Black, HPI – Hawaiian or Pacific Islander, and MR – Mixed Race; CIRS–G, Cumulative Illness Rating Scale for Geriatrics; PSWQ, Penn State Worry Questionnaire; HARS, Hamilton Anxiety Rating Scale; MADRS, Montgomery–Åsberg Depression Rating Scale; RSQ, Rumination Style Questionnaire; NEO–FFI, NEO–Five Factory Inventory; ERQ, Emotion Regulation Questionnaire; PSS, Perceived Stress Scale; Self–Worry Severity, average worry rating among worry statements rated as self–focused; Other–Worry Severity, average worry rating among worry statements rated as other–focused; Self–Worry Frequency, frequency of self–focused worry among worry statements; Worry Severity w/ Internal Reappraisal, average worry rating among reappraisal statements rated as internal locus of control (i.e., an index of effectiveness of internal reappraisal); Worry Severity w/ External Reappraisal, average worry rating among reappraisal statements rated as external locus of control (i.e., an index of effectiveness of external reappraisal); Internal Reappraisal Frequency, frequency of internal locus of control among reappraisal statements. All worry severity measures range from 1 (not worried at all) to 5 (extremely worried). Both frequency measures range from −1 (100% other/external) to 1 (100% self/internal)*.

This study employed a machine learning approach—elastic net regression to evaluate the association between each dependent variable: self-worry severity; other-worry severity; self-worry frequency; worry severity with internal reappraisal; worry severity with external reappraisal; and internal reappraisal frequency and the following predictor variables: age, sex, race, education, PSWQ, HARS, MADRS, RSQ, NEO-FFI, PSS, ERQ reappraisal, ERQ suppression, and CIRS-G. As some of predictor variables are correlated constructs (e.g., worry measured by PSWQ and neuroticism measured by NEO-FFI) [e.g., (45)], a standard linear regression model will encounter issues of multicollinearity, which inflates the variances of the parameter estimates. In order to avoid this, we employed elastic net regression, a regularized regression method, which is ideal to perform feature selection among the predictors that are highly correlated. Elastic net regression is an approach that solves the following regularization problem:


$$\frac{min}{ß}\left[\frac{1}{2N}\;*\sum_{i=1}^{N}{\left(y_{i}-x_{i}^{T}ß\right)}^{2}+\;\lambda \;*\sum_{j=1}^{p}\left(\frac{1-\alpha }{2}\;*\;{ß}_{j}^{2}+\alpha \;*\left|\;{ß}_{j}\right|\right)\right]$$

Where ß is the parameter estimate, N is the number of subjects, i is the i^th^ subject in the entire sample, y is the dependent variable, x is the independent variable or predictor, and j is the j^th^ predictor in the data. Along with the standard ordinary least squares regularization problem, we have an additional penalty term with λ representing the penalty per additional non-zero predictor in the data. Elastic net combines the L1 norm penalty (|ß|) of LASSO (least absolute shrinkage and selection operator) (46) with the L2 norm penalty (ß^2^) of ridge regression (47) by including a parameter α such that α = 1 reduces to LASSO, while α = 0 reduces to ridge regression. This approach overcomes issues with LASSO, primarily those related to small sample sizes and its bias toward selecting a single predictor among a set and ignoring others.

We used the ‘eNetXplorer’ package in R (48) to conduct six elastic net regressions for each dependent variable of interest. For each elastic net regression, the model ran for a set of 11 α values (0-1) and conducted 5-fold cross-validation to identify the value of λ (from a set of 50 pre-chosen values) that maximized the Pearson's correlation between actual dependent variable and the out-of-bag predicted dependent variable. This process was repeated over 500 runs. We then permuted the dependent variable and conducted this process again (250 permutations) to compute null bounds on the prediction of the model itself but also for the independent variables. This approach can estimate a *p*-value of the models as well as the *p*-value for each individual independent variable (48–50).

As a supplemental analysis, we also conducted six traditional multivariable linear regressions with each of the six dependent variables including all the independent variables. We have included these analyses in supplemental materials.

## Results

**Figure 1:**
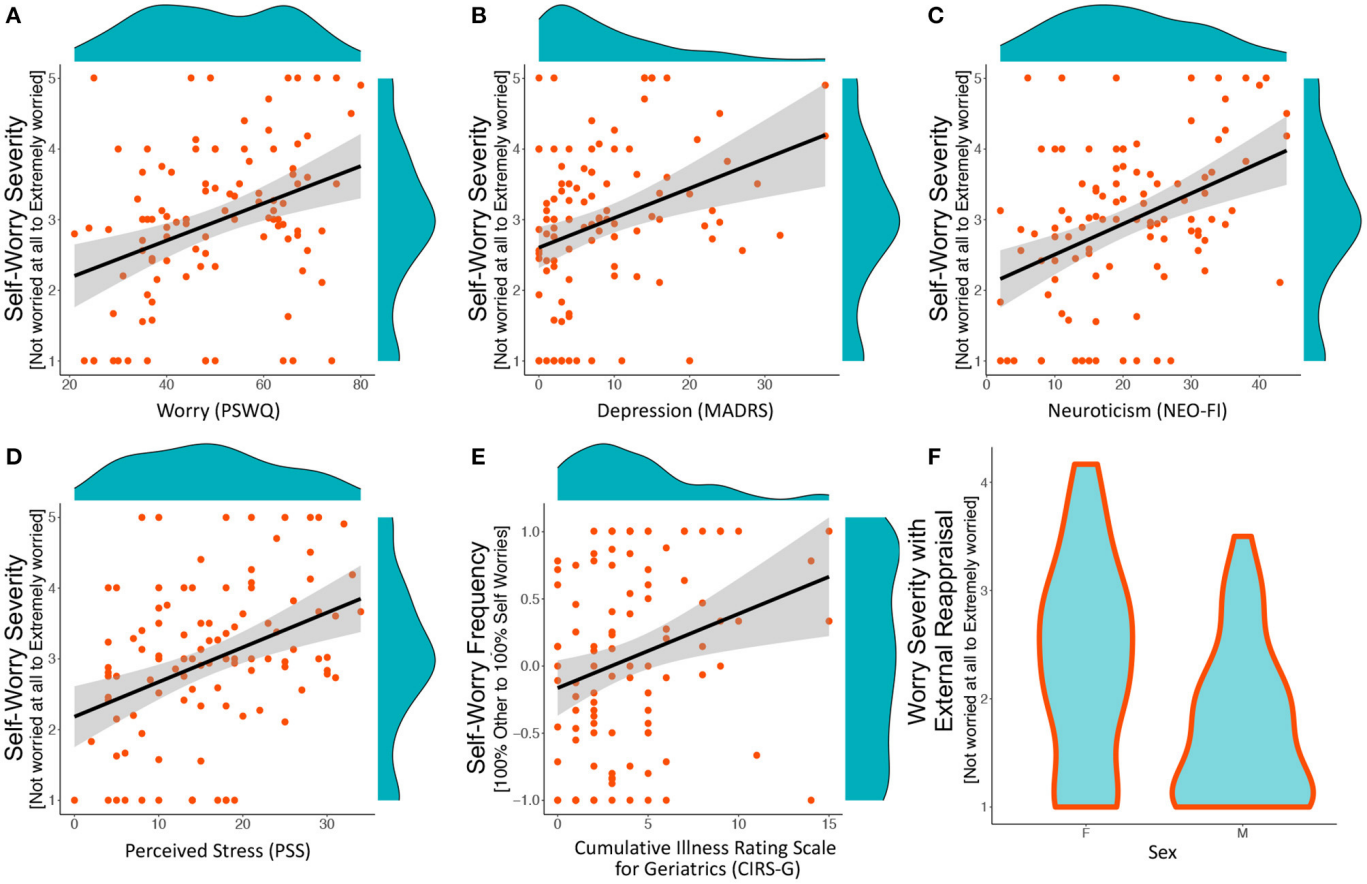
*Significant associations from elastic net analyses*. Plots A–E include a scatter plot, a least–squares line with 95% confidence intervals, and histograms in the margins. Plot F is a violin plot showing a mirrored histogram. **(A–D)** Associations between self–worry severity (average severity of self–focused worries) and clinical measures of worry, depression, neuroticism, and stress. **(E)** Association between self–worry frequency (frequency of self–focused worry among worry statements) classified as self–focused worries and CIRS–G (cumulative illness). **(F)** Violin plots showing differences between females and males on worry severity following external locus of control in reappraisal. This is a measure of worry severity after being shown the reappraising statements; therefore, higher values indicate greater worry despite being shown a reappraising statement.

The characteristics of the sample are reported in Table 1. PSWQ (a measure of worry) scores showed a moderately normal distribution (Figure 1A) with a mean of 50 (s.d. = 15), which is considered moderate worry (PSWQ moderate worry range is 40 to 59). Among 16 worry statements, slightly more than a half of statements were rated as “self” (coded as +1) (mean = 9.11, s.d. = 4.74), whereas the similar yet fewer number of statements were rated as “other” (coded as−1). On average, 1.81 statements were rated as “NA.” This resulted in having a close to zero score for Self-Worry Frequency (mean = 0.06, s.d. = 0.69). Among eight reappraisal statements, on average, approximately half of statements were rated as “self” (mean = 4.49, s.d. = 2.11), as well as “other” (mean = 4.40, s.d. = 1.95). On average, 1.25 statements were rated as “NA”. This resulted having a mean of 0.05 (s.d. = 0.59) for the Internal Reappraisal Frequency score. Both severity measures (Self-Worry Severity, Other-Worry Severity) showed similar degree of severity [mean = 2.97, 2.93, respectively). Similarly, both severity measures with reappraisal (Worry Severity w/ Internal Reappraisal, Worry Severity w/ External Reappraisal) showed similar degree of severity (mean = 2.28, 2.12, respectively).

We found that the following dependent variables were significantly predicted by the independent variables (i.e., out-of-bag prediction Pearson correlation was significant): self-worry severity (r_oob_ = 0.32, p_model_vs_null_ = 0.001, α_max_ = 0, λ_max_ = 2.79, r^2^ = 0.10), self-worry frequency (r_oob_ = 0.24, p_model_vs_null_ = 0.013, α_max_ = 0, λ_max_ = 1.18, r^2^ = 0.06); and worry severity with external reappraisal (r_oob_ = 0.24, p_model_vs_null_ = 0.026, α_max_ = 0, λ_max_ = 0.13, r^2^ = 0.06). The following were not significantly predicted by any of the independent variables: other-worry severity (r_oob_= 0.01), worry severity with internal reappraisal (r_oob_ = −0.08), and internal reappraisal frequency (r_oob_ = −0.03).

Greater self-worry severity (i.e., severity of worry on statements classified as “self” worry) was associated with greater general worry severity (PSWQ), depression (MADRS), neuroticism (NEO-FFI), and perceived stress (PSS) (Table 2, Figures 1A–D). Greater self-worry frequency (i.e., frequency of ‘self’ worries amongst the 16 statements) was associated with greater cumulative illness severity (CIRS-G) (Table 2, Figure 1E). Greater worry severity with external reappraisal (i.e., severity of worry after being shown a reappraisal that utilized an external locus of control) was associated with being female compared to male [i.e., women benefit less from reappraising statements that rely on others] (Table 2, Figure 1F). Standard multivariable regression models showed that same three dependent variables (self-worry severity, self-worry frequency, worry severity with external reappraisal) were predicted by the independent variables, which are reported in the supplement ( Supplementary Table S1).

**Table 2:** Significant predictors of self–worry severity, self–worry frequency, and worry severity with external reappraisal (only significant models).

	**Self–Worry**		**Self–Worry**		**Worry severity with**	
	**severity**		**frequency**		**external reappraisal**	
**Independent variable**	**_**weightedmean**_**	***p*** **–value**	**_**weightedmean**_**	***p*** **–value**	**_**weightedmean**_**	***p*** **–value**
Age	−0.06	0.053	−0.01	0.642	0.03	0.693
Sex [F ref]	−0.01	0.678	0.04	0.130	**−0.29**	**<0.001**
Education	0.01	0.656	−0.04	0.130	−0.01	0.943
Race [Non–White ref]	0.02	0.453	−0.02	0.533	0.12	0.142
Worry (PSWQ)	**0.06**	**0.016**	0.02	0.320	0.16	0.107
Anxiety (HARS)	0.03	0.253	0.03	0.156	0.07	0.484
Depression (MADRS)	**0.05**	**0.029**	0.02	0.216	0.05	0.629
Rumination (RSQ)	0.03	0.176	0.03	0.147	−0.15	0.113
Neuroticism (NEO–FFI)	**0.07**	**0.001**	0.03	0.114	0.12	0.248
Stress (PSS)	**0.06**	**0.005**	0.02	0.402	−0.11	0.252
Reappraisal (ERQ Subscale)	−0.04	0.155	−0.02	0.458	−0.05	0.567
Suppression (ERQ Subscale)	0.01	0.646	0.03	0.299	0.01	0.879
Cumulative Illness (CIRS–G)	0.01	0.819	**0.06**	**0.016**	−0.11	0.173

ß* weighted mean: Mean of feature coefficients (over runs) weighted by non–zero frequency (over folds). p–values (model vs. null) are generated by comparing to a null model with permuted dependent variables. Sex: female (F) was the reference group. Race: Non–White (i.e., Black, Hawaiian or Pacific Islander, or Mixed Race) was the reference group. Statistically significant results were listed as bold values*.

## Discussion

Greater severity of self-worry was associated with psychopathologic characteristics of anxiety disorders such as chronic worry (PSWQ), depression (MADRS), perceived stress (PSS), and neuroticism (NEO-FFI), while the severity of other-worry did not show any association with those measures in this sample. Greater frequency of self-worry (compared to other-worry) was not associated with the above-mentioned psychological measures, but was associated with a greater burden of physical illness (CIRS-G). In our analyses of reappraisal strategies, while internal locus of control in reappraisal appeared equally effective for both males and females, external locus of control in reappraisal was effective only for males. These results implicate that accessing the multiple aspects of self-referential processing may provide effective personalized therapeutic treatments. For late-life populations, clinicians should pay attention to the following components of worry: the object of worry (self vs. other), severity, and frequency since these reflect a different aspect of worry and may have a different treatment target. For example, severe self-worry (i.e., the self is the object of worry) may be the most malignant form of worry which needs attention with respect to their psychological burden. On the other hand, frequent self-worry may not be directly associated with psychopathological burden yet may reflect their worries stemming from their physical conditions. Therefore, intervention for physical or medical burden may be a therapeutic target. For older men, fostering an external locus of control as a reappraisal strategy may be an effective strategy to reduce their worry.

In anxiety disorders, distorted self-referential processing has been reported (16, 18) and may manifest as a form of excessive self-attention, extreme self-criticism, and self-deprecation. This self-referential processing profile may explain our results related to self-worry severity, indicating that an inner eye excessively focused on self-related worry scenarios correlates with multiple clinical markers of anxiety, depression, neuroticism and stress. Whether a consequence of the clinical categories or a premorbid trait, the self-referential processing profile is a common denominator of highly comorbid entities such as generalized anxiety, depression and neuroticism (16). The previous studies on self-focus in emotion processing indicated that some form of self-referential processing, particularly experiential self-focus (i.e., “How did I feel when the event happened?”), is adaptive, whereas evaluative self-focus (i.e., “Why did I have this problem?”) is maladaptive (51, 52). Our clinical samples of participants (mean PSWQ = 50) may spontaneously have recruited maladaptive forms of evaluative self-focus during worry induction. It is worth emphasizing that the reported severity for *other*-focused worry did not show any association with any of self-reported assessments. When the object of worry is about others, worry severity does not account for psychological burden. We may speculate that worrying about others may be a less malignant form of worry (e.g., a source of motivation to act on the issue) that does not directly contribute to psychological burden.

Among our self-reported assessments, some measures (i.e., anxiety by HARS, rumination by RSQ, reappraisal strategies by ERQ) did not show an association with self-worry severity in this sample. We speculate that HARS assesses the severity of broad anxiety related symptoms (e.g., insomnia, somatic symptoms) in GAD (53), whereas PSWQ items assesses specific aspects of worry. Rumination is a form of negative self-referential processing, but the target information processing may be different from worry. Rumination is a persistent negative thinking pattern focused on the past, whereas worry is concerned with potential future threats (54). We may speculate that the lack of association between severity of self-worry and reappraisal strategies as measured by ERQ may be related to the explicit nature of two regulation strategies measured by ERQ (cognitive reappraisal and expressive suppression). Most worriers use implicit strategies to decrease worry severity (55, 56), and self-worry severity may partially encapsulate the certain level of spontaneous implicit regulation during worry induction. In the analyses of self-worry severity, we also found the marginally significant age effect, suggesting that older participants reported less self-worry severity; however, our limited age range (50–80 years old) may yield a limited generalizability and may reflect a cohort effect.

As another index of self-referential processing in worry, we computed the frequency of self-worry (i.e., frequency of worry statements with the object of worry as “self” out of 16 statements) and found that greater self-worry frequency was associated with perceived physical impairments measure by CIRS-G. This frequency index was computed based on the personalized worry items that has varied levels of severity during the pre-scan interview. Therefore, the computed frequency reflects the prevalence of self as an object of worry among items that each participant worries about. Unlike self-worry severity, this frequency of self-worry was not associated with psychopathological burden but with physical illness burden (e.g., cardiovascular, endocrine, neurologic, and gastrointestinal) (38). Previous studies looking at the relationship between medical conditions and late-life GAD reported that individuals with GAD exhibited a significantly higher worry related to personal health (a combined measure of severity and frequency) than subclinical GAD and healthy individuals although the reported number of medical conditions were the same among groups (15). Our study extended this observation by using richer information of illness burden, taking severity of each condition into account, and identified more specific relationships between physical illness burden and worry. We may speculate that illness burden would increase frequency of how often self-worry occupies one's own mind; however, the higher frequency itself would not be a pathological form until self-worry (e.g., worrying about negative consequence of one's own illness) reaches a high degree of severity. This result may have clinical implications: frequent self-worry may be alleviated by effective interventions targeting physical conditions or by psychotherapeutic interventions addressing the role of medical burden.

We predicted that the external locus of control in reappraisal would be associated with psychopathologic characteristics of anxiety disorders (e.g., worry, depression) due to known anxiogenic effects of external locus of control (Hoehn-Saric and McLeod, [25, 26]). We expected that high frequency of external reappraisal compared to internal reappraisal would be associated with greater self-reported worry, depression, and stress. Contrary to our hypothesis, our results showed no association between internal reappraisal frequency and any of self-reported assessments nor demographic factors (age, sex, race, education). However, we found sex differences in the effectiveness of external reappraisal (i.e., worry severity with external reappraisal), while there were no sex differences in the internal reappraisal. Compared to women, men showed significantly lower worry severity when the corresponding reappraisal was utilizing an external locus of control. This sex difference might reflect the qualitative difference in the content of worry. While women with GAD tend to report more somatic related discomfort such as muscle tension, fatigue, gastrointestinal symptoms, men report interpersonal conflicts as a result of the excessive worry (57). If men and women worry about qualitatively different content, factors that may reduce worry might be also different for men and women. Our results may indicate that these factors which can reduce worry might be inherently external for men but not for women. The remaining question here is why external reappraisal was effective (only in men) against the known anxiogenic effect of external locus of control. Some studies suggest that acceptance of external resources that are outside of self-control in late-life plays an important role for “successful aging” (58–60). Gradually accepting and relying on external resources in late-life may be particularly challenging for women with GAD due to their more pronounced internalizing tendency, reflected in the higher rate of co-morbidity with internalized disorder (mood and other anxiety disorder), while men exhibit a higher prevalence of co-morbidity with externalized disorders such as substance abuse, conduct disorder, attention deficit hyperactivity disorder (ADHD), and antisocial personality disorders (57, 61, 62). Also, the observed sex difference may be stemmed from traditional gender-roles and gender-socialization such that women play a role of caregiver more than men throughout adult life (63). A transition from a caregiver to “successful aging” which requires accepting others' help may be psychologically taxing for women.

### Limitations

Due to the cross-sectional nature of the study, the directionality of the observed associations remains unknown. The present study aimed to examine self-referential processing among older individuals with various levels of worry. Our results from participants from only three decades in late-life have limited generalizability particularly to younger populations. Also, our transdiagnostic approach of worry allowed us to investigate individuals with low and high worry including a pathological range; however, future studies may need to confirm our results by using a dichotomous approach with a more uniform clinical group compared to a control group. Furthermore, we did not directly compare self-referential processing patterns between younger and older adults; therefore, we cannot conclude whether the reported self-referential characteristics were unique in late-life or not. Another limitation of the current study is a lack of a locus of control inventory (e.g., Rotter's Internal-External Control Scale) (20). We determined whether the reappraisal statements have an internal or external locus of control based on our own observations, which had high inter-rater reliabilities. Therefore, our index of locus of control assessment was specific to a reappraisal statement rather than general propensity of emotion regulation strategies (or attribution), or as a form of personality trait. Future studies should assess both specific (item-based) and general propensities regarding locus of control for emotion regulation strategies. Furthermore, we should emphasize that some cases of worries cannot be exclusively categorized into self-worry or other-worry since both types of worry can be fused. Worrying about a spouse's health could reflect one's own underlying worry about life after the loss of their spouse. In addition, it is worth noting that this was a secondary analysis of the behavioral data from the fMRI study with personalized statements. This dataset provided a unique opportunity to explore how multiple aspects of self-processing function in late-lite anxiety. As a future direction, it may be important to investigate the neural correlates of each self-processing index that we computed in the present study to enhance our understanding the neuropathology of late-life anxiety.

## Conclusions

The present study provides new insights regarding the self-referential thought processing associated with worry: higher self-worry severity was associated with various psychopathologic features, and higher frequency of self-focused worry was related to cumulative physical burden. We observed an interesting sex difference in effectiveness of reappraisal style in terms of locus-of-control: external reappraisal was effective only in men. Novel psychotherapeutic approaches, such as emotion regulation therapy (16) and rumination-focused cognitive-behavioral therapy (64), target altering distorted self-referential processing and have shown promising results. Acceptance and Commitment Therapy is another emerging approach that focuses on self-experience and mindfulness (65, 66), which is accumulating its effect particularly on late-life anxiety (67, 68). To further enhance these therapeutic approaches, future interventions and clinical practice may also explore these two types of expression of distorted self-referential processing, such as excessive self-focus, in higher degree of severity and frequency of worry about themselves, which are associated with different symptoms: psychological and physiological burden, respectively. Considering the sex differences in reappraisal style (i.e., an effectiveness of external locus of control in reappraisal only in men) may also be used for more personalized therapeutic interventions.

## Data Availability Statement

Data are available from the authors upon reasonable request and with permission of funding agencies.

## Ethics Statement

The studies involving human participants were reviewed and approved by University of Pittsburgh Institutional Review Board. The patients/participants provided their written informed consent to participate in this study.

## Author Contributions

AM, JN, HK, HA, and CA: study conception and design. HK, HA, and CA: data acquisition. AM, HK, JN, FK, MR, DM, HA, CA, and ML: analysis and interpretation of data. AM, HK, HA, CA, MR, and AN: drafting. AM, HK, DM, HA, and CA: critical revision. All authors contributed to the article and approved the submitted version.

## Funding

This work was funded by NIMH R01MH108509, NIMH R01 MH 076079, NIMH R01 MH121619, and NIA R01AG023651.

## Conflict of Interest

The authors declare that the research was conducted in the absence of any commercial or financial relationships that could be construed as a potential conflict of interest.

## Publisher's Note

All claims expressed in this article are solely those of the authors and do not necessarily represent those of their affiliated organizations, or those of the publisher, the editors and the reviewers. Any product that may be evaluated in this article, or claim that may be made by its manufacturer, is not guaranteed or endorsed by the publisher.
