# Supplementary material for: Thinking of Me or Thinking of You? Behavioral Correlates of Self vs. Other Centered Worry and Reappraisal in Late-Life
Source: Front Psychiatry. 2022 Jun 22;13:780745. doi: 10.3389/fpsyt.2022.780745 (PMC9256986; doi:10.3389/fpsyt.2022.780745)
Supplement: Supplementary file 1 [file Data_Sheet_1.pdf]

SUPPLEMENTAL MATERIALS

Thinking of me or thinking of you? Behavioral Correlates of Self vs. Other Centered Worry and Reappraisal in Late-life

Mizuno A.[1], Karim H.T.[1], Newmark J.[1], Khan F.[1], Rosenblatt M.[2], Neppach A.[3], Lowe M.[4], Aizenstein H.J.[1,2], Mennin D.S.[5], Andreescu C.[1]

[1]Department of Psychiatry, University of Pittsburgh, Pittsburgh, PA

[2]Department of Bioengineering, University of Pittsburgh, Pittsburgh, PA

[3]Department of Neuroscience, University of Pittsburgh, Pittsburgh, PA

[4]Department of Psychology, University of Pittsburgh, Pittsburgh, PA

[5]Department of Counseling and Clinical Psychology, Teachers College, Columbia University, New York City, NY

Author Notes

Correspondence concerning this article should be addressed to Carmen Andreescu, M.D. Western Psychiatric Hospital, 3811 O'Hara Street, Pittsburgh, PA 15213, United States. E-mail: andrcx@upmc.edu

Materials and Methods

We conducted six multivariable (multiple) linear regressions with each of the six dependent variables (self-worry severity; other-worry severity; self-worry frequency; worry severity with internal reappraisal; worry severity with external reappraisal; and internal reappraisal frequency) that we tested in the main analyses with the Elastic Net regression. We also included the same predictor variables: age, sex, race, education, PSWQ, HARS, MADRS, RSQ, NEO-FFI, PSS, ERQ reappraisal, ERQ suppression, and CIRS-G. All statistical analyses were conducted in R (R Core Team, 2013). We evaluated the regression to ensure that variance inflation factor < 5.

Results

The results showed that the following dependent variables were predicted by the independent variables: self-worry severity [F(13, 90) = 2.29, p =0.011, R^2 =0.25], self-worry frequency [F(13, 90) = 1.63, p =0.092, R^2 =0.19], and worry severity with external reappraisal [F(13, 90) = 2.28, p =0.012, R^2 =0.25]. The details of these results are summarized in Table S1. Other models with the rest of the dependent variables (other-worry severity, worry severity with internal reappraisal, and internal reappraisal frequency) were not significant.

Although the whole model with self-worry severity showed the statistical significance, none of the individual predictors predicted the self-worry severity. Greater self-worry frequency was associated with greater cumulative illness severity (CIRS-G). Greater worry severity with external reappraisal was associated with being female compared to male.

Table S1

Significant predictors of self-worry severity, self-worry frequency, and worry severity with external reappraisal (only significant models).

	Self-Worry Severity		Self-Worry Frequency		Worry Severity with External Reappraisal	
Independent Variable	*ß*	*p*-value	*ß*	*p*-value	*ß*	*p*-value
Age	-0.02	0.081	0.00	0.658	0.00	0.688
Sex [F ref]	-0.11	0.638	0.25	0.095	**-0.74**	**<0.001**
Education	0.02	0.623	-0.03	0.330	0.00	0.900
Race [Non-White ref]	0.39	0.203	-0.10	0.621	0.30	0.226
Worry (PSWQ)	0.00	0.841	0.00	0.907	0.01	0.122
Anxiety (HARS)	-0.04	0.149	0.01	0.624	0.01	0.621
Depression (MADRS)	0.04	0.119	0.00	0.981	0.01	0.716
Rumination (RSQ)	-0.01	0.338	0.01	0.493	-0.02	0.064
Neuroticism (NEO-FFI)	0.03	0.120	0.00	0.842	0.02	0.268
Stress (PSS)	0.02	0.286	0.00	0.823	-0.02	0.207
Reappraisal (ERQ Subscale)	-0.01	0.410	-0.01	0.591	0.00	0.764
Suppression (ERQ Subscale)	0.00	0.941	0.01	0.570	0.01	0.667
Cumulative Illness (CIRS-G)	0.01	0.823	**0.05**	**0.027**	-0.04	0.112

Note: Sex: female (F) was the reference group. Race: Non-White (Non-W: Black, Hawaiian or Pacific Islander, and Mixed Race) was the reference group.

References

Team, R.C. (2013). "R: A language and environment for statistical computing". (Vienna, Austria: R Foundation for Statistical Computing).
